# Psychometric properties of the Norwegian version of the Safety Attitudes Questionnaire (SAQ), Generic version (Short Form 2006)

**DOI:** 10.1186/1472-6963-8-191

**Published:** 2008-09-22

**Authors:** Ellen T Deilkås, Dag Hofoss

**Affiliations:** 1 Health Services Research Unit, Akershus University Hospital, Lorenskog, Norway

## Abstract

**Background:**

How to protect patients from harm is a question of universal interest. Measuring and improving safety culture in care giving units is an important strategy for promoting a safe environment for patients. The Safety Attitudes Questionnaire (SAQ) is the only instrument that measures safety culture in a way which correlates with patient outcome. We have translated the SAQ to Norwegian and validated the translated version. The psychometric properties of the translated questionnaire are presented in this article.

**Methods:**

The questionnaire was translated with the back translation technique and tested in 47 clinical units in a Norwegian university hospital. SAQ's (the Generic version (Short Form 2006) the version with the two sets of questions on perceptions of management: on unit management and on hospital management) were distributed to 1911 frontline staff. 762 were distributed during unit meetings and 1149 through the postal system. Cronbach alphas, item-to-own correlations, and test-retest correlations were calculated, and response distribution analysis and confirmatory factor analysis were performed, as well as early validity tests.

**Results:**

1306 staff members completed and returned the questionnaire: a response rate of 68%. Questionnaire acceptability was good. The reliability measures were acceptable. The factor structure of the responses was tested by confirmatory factor analysis. 36 items were ascribed to seven underlying factors: Teamwork Climate, Safety Climate, Stress Recognition, Perceptions of Hospital Management, Perceptions of Unit Management, Working conditions, and Job satisfaction. Goodness-of-Fit Indices showed reasonable, but not indisputable, model fit. External validity indicators – recognizability of results, correlations with "trigger tool"-identified adverse events, with patient satisfaction with hospitalization, patient reports of possible maltreatment, and patient evaluation of organization of hospital work – provided preliminary validation.

**Conclusion:**

Based on the data from Akershus University Hospital, we conclude that the Norwegian translation of the SAQ showed satisfactory internal psychometric properties. With data from one hospital only, we cannot draw strong conclusions on its external validity. Further validation studies linking the SAQ-scores to patient outcome data should be performed.

## Background

How to create a culture that supports patient safety is a question of considerable interest. Increasing efforts have been made to develop ways of measuring safety culture in clinical areas. Staff perceptions on workplace support for keeping patients safe emerges as an important measure. Safety culture surveys summarise staff perceptions on teamwork climate, safety climate, managerial support, self assurance, staffing and work environment factors. Results may be used to identify and help care-giving units that have problems with patient safety [1].

Implementing the comprehensive unit based safety program (CUSP) has been demonstrated to improve safety culture and reduce harm to patients [2]. CUSP consists of 8 steps; assessment of safety culture; sciences of safety education; staff identification of safety concerns; senior executives adopt a unit; improvements implemented from safety concerns; efforts documented and analyzed; results shared; and culture reassessed.

Patient safety culture can be studied quantitatively by surveys or qualitatively by anthropological/ethnographic methods – with a "middle category" consisting of questionnaires constructed to function as guidelines for reflective dialogue in staff groups, like the "Strategies for Leadership: an Organizational Approach to Patient Safety" (SLOAPS) [3], the "Checklist for Assessing Institutional Resilience" (CAIR) [4,5] and the Manchester Patient Safety Framework [6].

For quantitative surveys a number of questionnaires exist, including the "Hospital Survey on Patient Safety Culture" (HSOPS)[7], the "Veterans' Administration Patient Safety Culture Questionnaire" (VHA PSCQ) [8], the "Culture of Safety Survey" (CSS) [9] and the "Safety Attitudes Questionnaire" (SAQ)[10,11]. Reviews of a number of the most widely used quantitative safety culture survey instruments are presented by Colla, Bracken, Kinney and Weeks [12], and by Flin, Burns, Mearns, Yule and Robertson [13].

The purpose of this article is to present the psychometric properties of the generic version of the SAQ on Norwegian data. The version tested was the "Short Form 2006", containing 41 items and having separate response options for perceptions of management: "hospital management" and "unit management".

## Methods

### Data collection

#### Setting

The survey was carried out in the somatic clinical areas of Akershus University Hospital October-December 2006. The hospital has 500 somatic (and 200 psychiatric) beds, 4200 employees, and an annual budget of 2.500.000.000 NOK (approximately 450 million US$). It serves a population of 280 000 people, treats 53.000 in-patients and provides 150.000 out-patient consultations annually. Most in-patients (85%) are unscheduled emergency admissions. The safety culture survey was part of a patient safety strategy which the hospital has adopted, which follows guidelines developed by the Institute of Healthcare Improvement [14]. The heads of the clinical departments were informed about the survey in a meeting and in a letter from the CEO.

The study was approved by the Norwegian data inspectorate. We also applied for approval from the Regional ethics committee for medical research in Eastern Norway and they responded that our application was unnecessary because our study did not involve patients.

#### Questionnaire administration

Data were collected during regular staff meetings in the somatic care-giving units in agreement with the unit leaders, nurses by wards, physicians, physiotherapists and radiographers by department or section. Completing the questionnaire was voluntary. To alleviate fears of small-group responder identification, we promised that results would not be analysed across professions at unit level. Staff not present at the meetings was sent the questionnaire by hospital mail, with a preaddressed envelope and a sheet with information about the survey attached. To keep track of the number of questionnaires administered, questionnaires were numbered individually. The responders names were not recorded in the questionnaire and there were no name-and-number lists. Those who completed their questionnaire during the meeting were crossed out from the list of employees by the unit leader, who later told us who had not attended the meeting and should get their questionnaire by mail. Those who received it by mail crossed out their names on their local unit's list when they had returned the questionnaire. To reduce the number of non-responders, a designated person in the care-giving unit was asked to remind persons who hadn't crossed out their name in meetings and by poster.

Physicians and physiotherapists, who commonly work at more than one care-giving unit, were given the opportunity to fill out one questionnaire for each of up to three units. To keep account of the response rate the three questionnaires filled out by physicians and physiotherapists had the same number, but were supplied with an additional a, b, and c. Physicians and physiotherapists were asked to identify their care-giving unit and department, for other responders these boxes were filled out in advance. The information sheet contained a list of care-giving units participating in the study.

Questionnaires were distributed to 1911 frontline personnel in 47 somatic care giving units of 14 ambulatory clinics, 27 wards, four labs, one operation unit, and one anaesthetic department. 762 staff were given the SAQ in staff meetings and 1149 received it through the hospital's postal system.

### The Safety Attitudes Questionnaire

#### Development and History

The Safety Attitudes Questionnaire is a further development of the Intensive Care Unit Management Attitudes Questionnaire [15,16], originally derived from the FMAQ [17], a traditional human factors survey with a 20-year history in aviation [10]. The SAQ consists of items both from the FMAQ and new items generated on the basis of Vincent's framework for analysing risk and safety [18] and Donabedian's conceptual model for assessing quality [19].

The items were evaluated through pilot testing and exploratory factor analysis which led to identification of the following six factors; safety climate, teamwork climate, stress recognition, perceptions of management, working conditions and job satisfaction.

#### Items and Factors

The SAQ has been adapted for use in ICUs, operating rooms, inpatient wards, ambulatory clinics, emergency departments, maternity wards, and pharmacies. it also exists in a generic version where the care-giving areas are not specified in the items like in 'Nurse input is well received in this a ICU' but instead kept neutral like in 'Nurse input is well received in this care-giving area'. A short form version is also made where six additional items are included together with 30 items belonging to the six factors. The additional items were added because they were considered interesting in their own right to senior leaders participating in the pilot studies [10]. The items belonging to each factor are listed in Additional file 1. For our study we translated a short form generic version.

#### Scales and Scoring

The score of the factor scales may be calculated by doing the following. First the results of negatively worded items (2 and 11) must be reversed. One is subtracted from the mean of the set of items from the scale, and the result is multiplied by 25. The percentage of respondents who "agree slightly" or "agree strongly" for each of the items within a factor are charted as the percent positive for each SAQ factor.

#### Evidence on SAQ data validity and reliability

The SAQ is probably the best documented instrument for measuring patient safety culture [10,12]. Benchmark scores from 203 clinical areas in USA, UK and New Zealand have been published with an overall response rate of 67%, ranging from 66% to 72% across administrations [10]. Incomplete data at item level was approximately 1.5% overall, with a range between 0.3–3.5%. Multilevel confirmatory factor analysis gave a χ^2 ^(784) = 10311.27, p < 0.0001; CFI = 0.90 and RMSEA = 0.03. Composite scale reliability was assessed via Raykov's *ρ *coefficient and was 0.90, which indicates strong reliability.

The SAQ is also the only questionnaire which shows links to patient outcome: a well-developed patient safety culture, as measured by the SAQ, has been shown to correlate with fewer medication errors, lower ventilator associated pneumonia rates, fewer blood-stream infections, and shorter ICU lengths of stay [1,12].

The SAQ is the most widely used instrument for measuring patient safety culture. Including our Norwegian translation the SAQ has now been translated into seven languages, and has been administered in over 1300 hospitals in the USA, United Kingdom, Switzerland, Germany, Norway, Sweden, Spain, Portugal, Italy, Turkey, and New Zealand (written communication, C. Fullwood, Oct 2007).

#### The translation into Norwegian

Linguistic validation of our translation was performed with the back-translation technique [20]. The questionnaire was first translated from English into Norwegian by one translator and then translated back into the source language by an independent translator (an American nurse and researcher who has worked for many years in Norway and is fluid in both languages), who was blinded to the original questionnaire. We (ED and DH) compared independently the instrument in its original English version and the version translated back to English, and discussed the retranslation with one of the authors of the American questionnaire, resulting in minor reformulations of the translation of a small number of items before the Norwegian version of the questionnaire was tried out at the Akershus University Hospital.

### Statistical analysis

#### Data quality

Missing at item level are shown in Additional file 1 and was on average 2.9%, within a range of 0 to 13%.

The table also shows means and standard deviations for each item. Item responses were clearly skewed towards the positive, but showed considerable variation For all items, all categories were ticked [Additional file 1].

**Figure 1:**
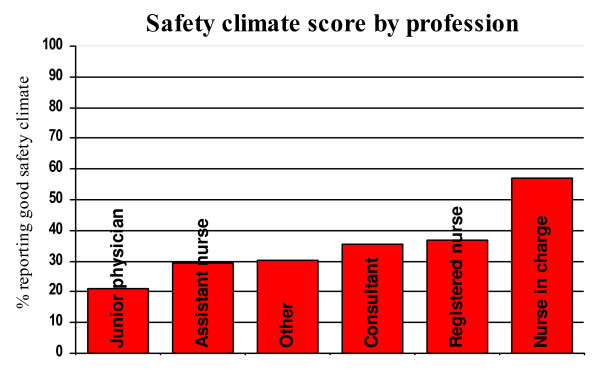
Variation in Safety climate factor average across professions.

**Figure 2:**
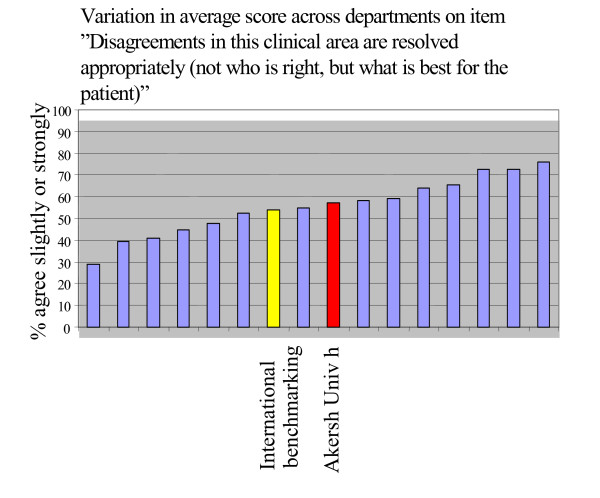
Variation across departments in average score on item "Disagreements in this clinical area are resolved appropriately (not *who *is right, but *what *is best for the patient").

**Figure 3:**
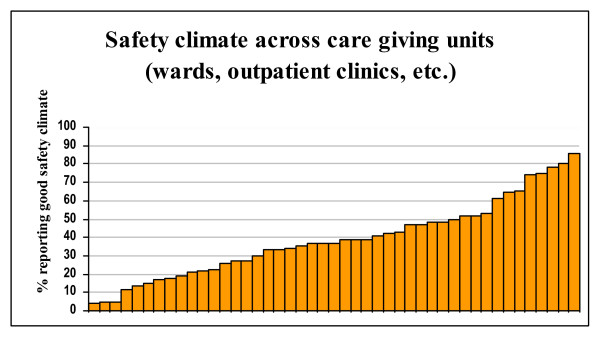
Variation in Safety climate factor average across wards/outpatient clinics.

There was also considerable variation across professions, departments, and – particularly – wards, as exemplified by Figures 1, 2 and 3.

#### Data processing

Questionnaires were scanned by the optical reading program Snap Survey. In cases where different postal responders had used different names for the same care-giving unit (for example "S5" and "Big children ward"), we harmonized the names into a complete and mutually excluding list of unit names. The confirmatory analysis was done by AMOS. SPSS was used to estimate Cronbach alphas, item-to-own correlations, intercorrelations of factors, test-retest correlations and all item-descriptive statistics.

#### Confirmatory factor analysis: internal construct validity

The factor structure of the responses were analysed using AMOS, a program that performs confirmatory analysis (CFA). CFA is the form of factor analysis which provides formal tests of the goodness of the fit of the pre-hypothesised factor model to the data. We report these goodness-of-fit indices: the chi square, the chi-square/df-ratio, the p, the p_close_, the Adjusted goodness-of-fit index (AGFI), the Root mean square error of approximation (RMSEA) and the Hoelter 0.05. Acceptable goodness of fit-values indicate internal construct validity of the model – in this case, that what the questionnaire measures is patient safety culture expressed in the hypothesised factors. Suggested criteria values are chi-square not exceeding the number of degrees of freedom of the model, although Wheaton & al [21] suggests accepting any chisquare/df-ratio under 5, and Carmines and MacIver [22] consider values of 2–3 acceptable, whereas Byrne [23] will not accept ratios above 2. The p and p close values should exceed .05 [24], although Jöreskog [25] cautions that large samples may preclude such low p-values even in good models – which is why the Hoelter 0.05 [26] (an estimate of the largest sample for which a data set with these intercorrelations among the variables would confirm the model) should exceed 200. The Adjusted Goodness of Fit Index should be close to 1 – but most AGFIs are, and it is not clear which lower values speak against the model. The Root Mean Square of Approximation (RSMEA) should not exceed 0.10 [24].

#### Internal consistency

The internal consistency of the factors was assessed by item-total correlations, checking that all items were more highly correlated with the factor they were hypothesised to belong to than with any other factor, and by Cronbach alphas (consistent factors should have alphas exceeding 0.7 [27].

The test-retest reliability was assessed in the hospital's radiology lab, which with its 97 employees is one of the largest clinical units in the hospital. Its questionnaires were, in addition to the serial number, marked to show if the questionnaire was from the first measurement or the second. The time interval between the two measurements was three weeks. Test-retest stability was assessed by the intraclass correlation coefficient, which should exceed 0.7 [28].

#### Hypothesised factor structure

Technical reports from the SAQ developers at the University of Texas at Austin and the Johns Hopkins University specifies the six factors as described in Additional file 1 and Table 1[11]. No report on the factor structure of the generic short version of the SAQ has been published. As this version of the questionnaire introduced a split of the questions on perceptions of management into two sets, one on hospital (top) management and one on local (unit) management, we were obliged to reformulate slightly the questionnaire's hypothesised factor structure by imagining two management perception factors instead of one, each containing one set of the five split questions on perceptions of management, as shown in Additional file 2. Because the SAQ Short Form item #29 ("The levels of staffing in this clinical area are sufficient to handle the number of patients") were not included in the items split on unit and top management, we concluded it was not considered a part of the two perceptions of management factors. We hypothesised it to be part of the working conditions factor. In our hypothesised seven factor structure, factors Teamwork climate, Safety climate, Stress recognition, and Job satisfaction are identical with the ones defined by the developers of the non-generic SAQ-versions (except that "this clinical area" was substituted for "this ICU"). The three other factors were hypothesised as in Table 2.

**Table 1:** The six SAQ factors of the non-generic SAQ versions (ICU version)

Teamwork climate	Nurse input is well received in this ICU
	In this ICU, it is difficult to speak up if I perceive a problem with patient care
	Disagreements in this ICU are resolved appriopriately (i.e. not *who *is right, but *what *is best for the patient)
	I have the support I need from other personnel to care for patients
	It is easy for personnel in this ICU to ask questions when there is something that they do not understand
	The physicians and nurses here work together as a well-coordinated team
Safety climate	I would feel safe being treated here as a patient
	Medical errors are handled appropriately in this ICU
	I know the proper channels to direct questions regarding patient safety in this ICU
	I receive appropriate feedback about my performance
	In this ICU, it is difficult to discuss errors
	I am encouraged by my colleagues to report any patient safety concerns I may have
	The culture in this ICU makes it easy to learn from the errors of others

Stress recognition	When my workload becomes excessive, my performanced is impaired
	I am less effective at work when fatigued
	I am more likely to make errors in tense or hostile situations
	Fatigue impairs my performance during emergency situations (e.g. emergency resuscitation, seizure)

Working conditions	This hospital constructively deals with problem physicians and employees
	This hospital does a good job of training new personnel
	All the necessary information for diagnostic and therapeutic decisions is routinely available to me
	Trainees in my discipline are adequately supervised

Job satisfaction	I like my job
	Working in this hospital is like being part of a large family
	This hospital is a good place to work
	I am proud to work ast this hospital
	Morale in this ICU area is high

Perceptions of management	Hospital management supports my daily efforts
	Hospital management does not knowingly compromise the safety of patients
	I am provided with adequate, timely information about events in the hospital that might affect my work
	The levels of staffing in this clinical area are sufficient to handle the number of patients

**Table 2:** Re-hypotesizing three SAQ factors for the generic SAQ version

Perceptions of hospital (top) management	Hospital management supports my daily efforts
	Hospital management doesn't knowingly compromise patient safety
	Hospital management is doing a good job
	Problem personnel are dealt with constructively by our hospital management
	I get adequate, timely information about events that might affect my work from hospital
	management
Perceptions of unit management	Unit management supports my daily efforts
	Unit management doesn't knowingly compromise patient safety
	Unit management is doing a good job
	Problem personnel are dealt with constructively by our unit management
	I get adequate, timely information about events that might affect my work from unit management

Working conditions	This hospital does a good job of training new personnel
	All the necessary information for diagnostic and therapeutic decisions is routinely available to me
	Trainees in my discipline are adequately supervised
	The levels of staffing in this clinical area are sufficient to handle the number of patients

#### SAQ external validity

Our data set did not include data on patient (un-)safety that could be related to our SAQ-scores. We were, however, given access to two other data sets, collected at the same time and at the same hospital (if only at a few clinical departments), which described patients' evaluations of the organization of the hospital work, patients' suspicion of having possibly been maltreated, and patient record documentation of adverse events [29]. We have therefore been able to correlate our SAQ-scores with the average department patient satisfaction scores (in 4 departments) and with the departments' percentage of patient records containing indications of adverse events (in 6 departments). The low number of departments will not allow any positive conclusions, but a lack of correlation with their SAQ-scores could be considered a sign of low external validity.

## Results

### Response rates

1306 of the 1911 persons invited to participate completed and returned the questionnaire (68%). Including the additional questionnaires returned by physiotherapists and physicians who served more than one ward, a total of 1460 completed questionnaires were returned. The response rate was much higher for questionnaires distributed in meetings (96%) than for those distributed through the mailing system (50%). The response rate was markedly lower for physicians (52%) than for non-physicians. The response rate varied across units from 44% to 100%.

### Item-to-total correlations

All items correlated more highly with its own factor than with any other factor as shown in Additional file 1.

### Cronbach's alphas

The Cronbach's alphas (0.68 to 0.85) of our seven factors are shown in Additional file 1. For no factor the exclusion of any variable would noticeably increase the α-value.

### Test-retest reliability

Test-retest intraclass correlation coefficients were considerably higher for (additive) factor scores (reversed items were re-reversed before summing) than for single items as shown in Additional file 1, for five of the seven factors test-retest intraclass correlation coefficients exceeded 0.7 (the exceptions were Stress recognition and Perceptions of hospital top management).

Correlations were considerably higher among physicians than among other staff, both for single items and for (additive) factors – for physicians, all intraclass correlations, except for factor Stress Recognition (0.67) were above 0.7.

### Construct validity: goodness of fit values for the confirmatory factor analysis model

**Figure 4:**
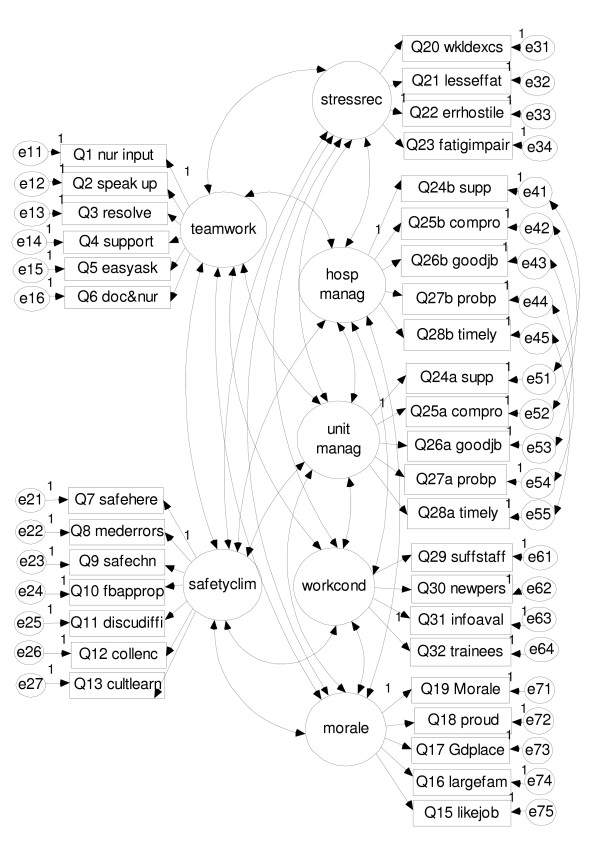
Factor structure model.

We tested the factor structure by confirmatory factor analysis. Our factor structure model is presented in Figure 4.

Goodness-of-fit indices for the model are shown in Table 3.

**Table 3:** Goodness-of-fit indices for factor structure model

	Entire model, viewed as a whole (n = 696)	Team-work climate (n = 1082)	Safety climate (n = 0999)	Stress recognition (n = 1039)	Perception of hospital management (n = 922)	Perception of unit management (n = 963)	Working conditions (n = 952)	Morale (n = 1051)
χ^2^/d.f.	2.583	6.896	15.923	59.014	2.591	6.373	2.263	6.49
p	< .001	< .001	< .001	< .001	.024	< .001	.104	< .001
p_close_	.893	.012	< .001	< .001	.646	.042	.616	.051
AGFI	.871	.955	.869	.718	.983	.963	.988	.964
RMSEA	.048	.073	.122	.236	.042	.075	.036	.072
Hoelter .05	296	301	107	53	788	335	1259	359

### Early external validation

Two indications provide early external validation of the translation used at the Akershus University Hospital. In January-May 2007 the Akershus University Hospital's Quality Department checked the records of a random sample of 481 patient journals in four of the hospital's departments by the Global Trigger Tool Method advocated by the Institute of Healthcare Improvement [30]. The departments' percentage of patients whose records document that they experienced an adverse event during hospitalization correlated strongly (except for the factor Stress recognition) with the departments' average staff SAQ-factor scores, as shown in Table 4 – of course, due to the very low number of departments studied, only one of the correlation coefficients was significant at the 0.05-level.

**Table 4:** Correlation of average department staff SAQ-scores with department fraction of patient records suggesting an adverse event took place during hospitalization (N = 4)

Average department staff score on teamwork climate:	-0.99 (p < .01)
Average department staff score on safety climate:	-0.93 (n.s.)
Average department staff score on stress recognition:	-0.08 (n.s.)
Average department staff score on perceptions of hospital management:	-0.77 (n.s.)
Average department staff score on perceptions of unit management:	-0.93 (n.s.)
Average department staff score on working conditions:	-0.92 (n.s.)
Average department staff score on job satisfaction:	-0.91 (n.s.)

Also, as shown in Additional file 3, the average SAQ-scores of staff of six departments at the Akershus University Hospital correlated with the average scores of 178 randomly chosen patients on questions on possible maltreatment, perceived clumsiness of hospital work and general satisfaction with hospitalization, collected (by the Norwegian Knowledge Centre for the Health Services, which has provided the department average patient scores used to produce additional file 3) at the same period of time at the same departments.

## Discussion

Because we personally visited all hospital units to collect the data, we could observe that the questionnaire was met with interest – but generally with less enthusiasm from physicians than from others. The questionnaire was not regarded as threatening. Only two units of the 49 approached declined the invitation to participate, and only one of them because it did not want to go on record at this moment, the other was a laboratory unit which found the generic patient safety questionnaire irrelevant to their tasks. The response rate was relatively high (68% – among physicians, however, only 52%), and, as shown in Additional file 1, very few items produced a large number of missing responses. The outstanding exception was "I experience good collaboration with pharmacists in this clinical area", which had a missing rate of 20%. In our hospital, pharmacists do not participate in daily procedures in care-giving areas; their cooperation with the units is limited to more or less annual inspections. The reason why many have not responded to this item is probably that they found it irrelevant.

A number of respondents asked how to understand the item "Fatigue impairs my performance during emergency situations (e.g. emergency resuscitation, seizure)". Their comments have convinced us that the translation into Norwegian of this item should be reformulated and should not read "Slitenhet reduserer måten jeg opptrer på i krisesituasjoner (som resuscitering, anfall o.l.)" but "Jeg arbeider dårligere i krisesituasjoner (som resuscitering, anfall o.l.) når jeg er sliten".

The questionnaire was not very time-consuming. In all clinical units at the Akershus University Hospital we observed that most responders completed the questionnaire within the 10–15 minutes suggested by the SAQ technical reports[11], and all respondents finished within 20–25 minutes.

A data collection challenge was to ensure that all those who participated in patient care at the care-giving units were invited to participate in the data collection. The problem was that many physicians and physiotherapists were not employed by any specific unit and therefore did not attend unit staff meetings. These caregivers had to be reached in their own professions' group meetings.

The relatively high response rate, low number of missing data and the relatively short completion time testify to the acceptability of the SAQ in the Norwegian setting. One item, however, stood out as a candidate for removal, since not many Norwegian clinical workers cooperate directly with pharmacists – in fact, one may wonder why not many more than 20% of the responders left the question of the quality of their cooperation with pharmacists unanswered.

Responses were, for most – but not all – items skewed towards the positive end of the scale. But the response distributions did not suggest that any particular item or set of items should be removed for failing to reflect variation.

All items were, as they should be, more strongly correlated with their own factor than with any of the others.

The relatively high Cronbach alphas for all hypothesised factors demonstrates the internal consistency of the factors: all alphas were between 0.71 and 0.85 – except for the factor Teamwork climate, but its alpha of 0.68 was not much below the recommended limit of 0.70.

The stability of the questionnaire also proved acceptable: the test-retest intraclass correlation coefficients of the factors were relatively high – except for factors Stress recognition (0.55) and Perceptions of hospital management (0.44). A possible interpretation is that in the average clinical worker's eyes, the hospital's top management is so distant that it is difficult to maintain a stable perception of its qualities. The fact that the test-retest correlation for Perceptions of hospital management was practically zero for non-physicians, but quite high (0.83) for physicians lends credibility to that interpretation. The relatively low retest stability of the Stress recognition score, too, was due to the low correlation for non-physicians, whose stress load may feel much more variable and beyond control than the physicians'. The striking difference in the three-week test-retest intraclass correlation coefficients between physicians and others may indeed be seen as suggesting that checking a questionnaire's reliability by the stability of the responses to it is more appropriate among staff who are likely to feel reasonably in command of their work. The items made no reference to the length of the period to be taken into consideration when ticking the questionnaire, and for those more easily subject to the variable demands of those higher in the hospital hierarchy, work must be expected to be appear more variable. Users of the Norwegian translation might want to double-check the test-retest reliability of this factor, and interpret this factor score with due regard to its stability.

The construct validity of the questionnaire, as judged by the goodness-of-fit indicators from the confirmatory factor analysis, can be considered acceptable, but less than perfect. Some of the goodness-of-fit indices speak against the fit of the model to the data, namely the p-value of less than 0.001 and the AGFI of 0.871. But the χ^2^-value (2.583) was within the limits indicated by Wheaton et al [21]and Carmines and MacIver[22]. And the p_close _(0.893) and the RMSEA (0.048) both exceeded the criteria suggested by Browne and Cudeck [24], and the Hoelter 0.05-value of 296 was above the critical value given by Hoelter[26].

The questionnaire cannot be regarded as externally validated until more hospitals have been surveyed and the results from similar units can be compared and related to patient outcomes. However, our informal impression from our feeding the results back to the clinical units and from our presentation of the results to the hospital's top management and to its Quality department is that the responding units seemed to feel not surprised by their SAQ-scores, and that the hospital top management and Quality department felt the scores were credible. Department average scores also correlated with the frequency of adverse hospital events (as determined by Global trigger tool revision of patient records) and with department average patient reports on general satisfaction with hospitalization, worries about possible maltreatment, and evaluation of the smoothness of hospital work.

## Conclusion

On the basis of the above evidence, we conclude that the Norwegian translation of the generic short-form version of the Safety Attitudes Questionnaire is a reasonably reliable and possibly also valid instrument for the measurement of patient safety culture in hospitals.

From our test experience we would, however, like to suggest two minor adjustments. First, comments from the respondents at Akershus University Hospital showed that our translation into Norwegian of the item "Fatigue impairs my performance during emergency situations (e.g. emergency resuscitation, seizure)" should be reformulated as shown above, and listed in Additional file 2. Second, the question on cooperation with pharmacists might be considered for removal from the Norwegian version: very few Norwegian clinical workers cooperate directly with pharmacists.

Finally, one should be aware that the generality of the generic SAQ version is threatened by the word "nurse", which may alienate radiographers, laboratory technicians, secretaries, physiotherapists etc.

## Competing interests

The authors declare that they have no competing interests.

## Authors' contributions

Both authors contributed to the translation of the SAQ into Norwegian, data collection and analysis and the writing of the report. Both authors have read and approved the final manuscript.

## Pre-publication history

The pre-publication history for this paper can be accessed here:



## Supplementary Material

Additional file 1 **Item variation, internal consistency and test-retest reliability (ICC) by factor).** Click here for file

Additional file 2 **SAQ 2006 Short form, original formulations, Norwegian translations.** Click here for file

Additional file 3 Correlation of average department staff SAQ-scores to average department patient scores on (response scale 1–5) variables* "General satisfaction with hospitalization", "Maltreatment suspicion", and "Hospital work organization" (N = 6). *"General satisfaction with hospitalization" = "All things considered, were you generally satisfied with hospital treatment and care?" "Maltreatment suspicion" = "Do you feel that you were in any way maltreated (as far as you are able to judge)?" "Hospital work organization" = An index built from the answers to three questions: "Was it your impression that you were cared for by a permanent group of nursing staff?", "Was it your impression that one doctor were responsible for you?", and "Was it your impression that hospital work was well organized?". Click here for file
